# Elite athletes: what lessons for the future can be learned from their responses to the social disruption caused by the COVID-19 pandemic?

**DOI:** 10.3389/fspor.2023.1260797

**Published:** 2023-11-02

**Authors:** Kaja Poteko, Jay Coakley, Mojca Doupona

**Affiliations:** ^1^ Department of Social Sciences and Humanities in Sport, Faculty of Sport, University of Ljubljana, Ljubljana, Slovenia; ^2^ Sociology Department, University of Colorado, Colorado Springs, CO, United States

**Keywords:** elite sport, elite athletes, COVID-19 pandemic, guidelines, social disruptions

## Abstract

In mid-March 2020, the Covid-19 pandemic was declared, disrupting established routines and impacting every aspect of our lives. Sport as a social phenomenon was no exception. On the one hand, with the suspension and postponement of competitions and the various restrictions, the pandemic seemed to stop time, destroying the previously familiar ways of functioning of the sports sector, and forcing it to constantly reinvent, restructure and adapt. On the other hand, the changed situation highlighted the problems and inequalities that sport had faced long before and that the pandemic had mostly exacerbated, but not caused. In this review article, we identify the lessons and insights the pandemic has brought, especially for elite athletes. Because many scientific articles have emerged in connection with the pandemic, we select and review them according to our focus on elite athletes and summarise their findings. We then use those findings to derive guidelines that can serve top athletes as a tool in potentially similar situations in the future. Our proposed guidelines are divided into macro-, meso-, and micro-social levels, encompassing national and international, organizational and individual perspectives.

## 1. Introduction

In mid-March 2020, the World Health Organization declared an international public health emergency or pandemic Covid-19, caused by the severe acute respiratory syndrome coronavirus-2. From the outset, it was clear that the pandemic was far more than a global health problem and that we were dealing with a crisis at multiple levels of society. On the one hand, the Covid-19 pandemic, which superficially appeared to be only a health crisis, also impacted other levels of daily life, affecting the social, cultural, environmental, economic, and political spheres of social life (1). Through the disruption of “business as usual”, our familiar routines and everyday life, social relationships and social institutions were suspended, restored and/or reconfigured [(1), p.11].

On the other hand, the pandemic also offered us some insights into the existing organization of social life, and for understanding and dealing with this pandemic, much could be learned from the literature that focuses on how societies have responded to previous epidemics and pandemics (2). As noted in the past, pandemics can serve as a mirror of society. Aspects of society that are otherwise taken for granted or hidden, such as social inequalities and social exclusion, often come to the fore (2) and we “cannot register a global phenomenon such as a pandemic without at once registering those inequalities” and seeing them intensify [(3), p.2]. The Covid-19 pandemic was no exception in this regard—while it spread in waves that phenomenologically correlated with hope and despair (3), wherever the virus struck it revealed existing inequalities and class divisions inscribed in our societies (4). If on the one hand we often encountered the slogan that “we are all in the same boat”, on the other hand we found that while we all had a problem and the virus could infect anyone, we dealt with the situation and its consequences very differently. In other words, even if the virus did not discriminate, it was the social system itself that did (4).

Sport, as an important social sphere, was no exception in this context. The pandemic touched all aspects of both elite and recreational sport and, as a social phenomenon, affected, albeit in different ways, those who actively participate in sport as well as those who follow it as consumers, those who manage it in one way or another through various organizations, clubs, and institutions, those who shape sports policy, those who report on it, those who invest in it, those who research it, and so on. In the context of elite sport, the pandemic first and most visibly disrupted all sporting events and leagues, leading to the cancellation or postponement of various competitions (from the smallest to mega sporting events, e.g., the postponement of the Tokyo Olympics). Then, when sporting events resumed, the changed social situation altered both the previously established forms of active participation in sport and the forms of its consumption, bringing with it some unusual and unprecedented features, such as the silence in the stadiums with the absence of spectators at sporting events.

As a social situation that suspended and restored social relations, the pandemic in the context of sport brought to the surface various discourses and debates about the social role of sport, various sport stakeholders wondered how they could restore it, and sport actors sought ways to maintain the relevance of sport (5, 6–19). For elite athletes, the general uncertainty and not knowing if or when competitions would take place made training much more difficult and achieving peak performance was undermined. Access to certain training venues deteriorated, making it difficult to maintain continuity of training (11, 12, 14). At the same time, the contagion of the virus, especially in the early stages of the pandemic, forced athletes to interrupt their training. Cancelation of competitions affected qualification for future competitions. Advertising contracts, sponsorships, and salaries were negatively affected, and some athletes lost their jobs and were financially insecure (7–10). In addition, the lack of training support from teammates negatively impacted some athletes’ motivation to train, which was exacerbated by the decrease in personal contact with coaches.

The pandemic officially lasted 3 years, alternating between periods that were more restrictive on the level of general social life, depending on the time and space, and periods that were more relaxed in this respect. It was also during this period that its impact on sports and athletes became an important topic and the subject of numerous researchers and research projects and reflections in academia that attempted to capture both the more naturalistic and the more social and humanistic aspects of the pandemic. Today, when we look in various scientific databases to see how much scientific work and academic attention the pandemic has attracted, we come across extraordinary numbers, exclusively in the form of scientific articles, commentaries, editorials and similar texts (the methodological part of this review shows almost 11,000 articles that have appeared exclusively in connection with our search terms!). In this respect, it can therefore be important and useful to review the diverse findings, results and contributions of different scholars and try to present and possibly synthesize them in a more condensed form.

In this paper, we set ourselves a similar task, as we are interested in what we can learn from this pandemic and how we can possibly transfer and apply some of the lessons to other, possibly similar, social circumstances in the future. More specifically, we focus on elite sports and elite athletes, assuming as a criterion that athletes compete at least at the national level and derive guidelines that might help them in potentially similar situations. In other words, we are interested in the impact of the coronavirus pandemic on different aspects of elite/professional athletes’ lives from micro- (individual), meso- (organizational), and macro-social (national and international) perspectives. How has the pandemic affected them? How did they (un)successfully adapt to the new situation? What tools and mechanisms did they use and what social circumstances enabled them to do so? What lessons and habits have they acquired that are transferable and could make it easier for them to handle similar social situations in the future? In the continuation of the paper, we first explain at the methodological level the process of searching for academic papers that fit the context of our efforts, explain how we arrived at the final selection of articles suitable for review, and then present their findings and also synthesize them by deriving guidelines. In this way, we provide elite athletes with a guide for their responses and coping strategies in the context of similar crisis situations in the future.

## 2. Methodology

**Figure 1:**
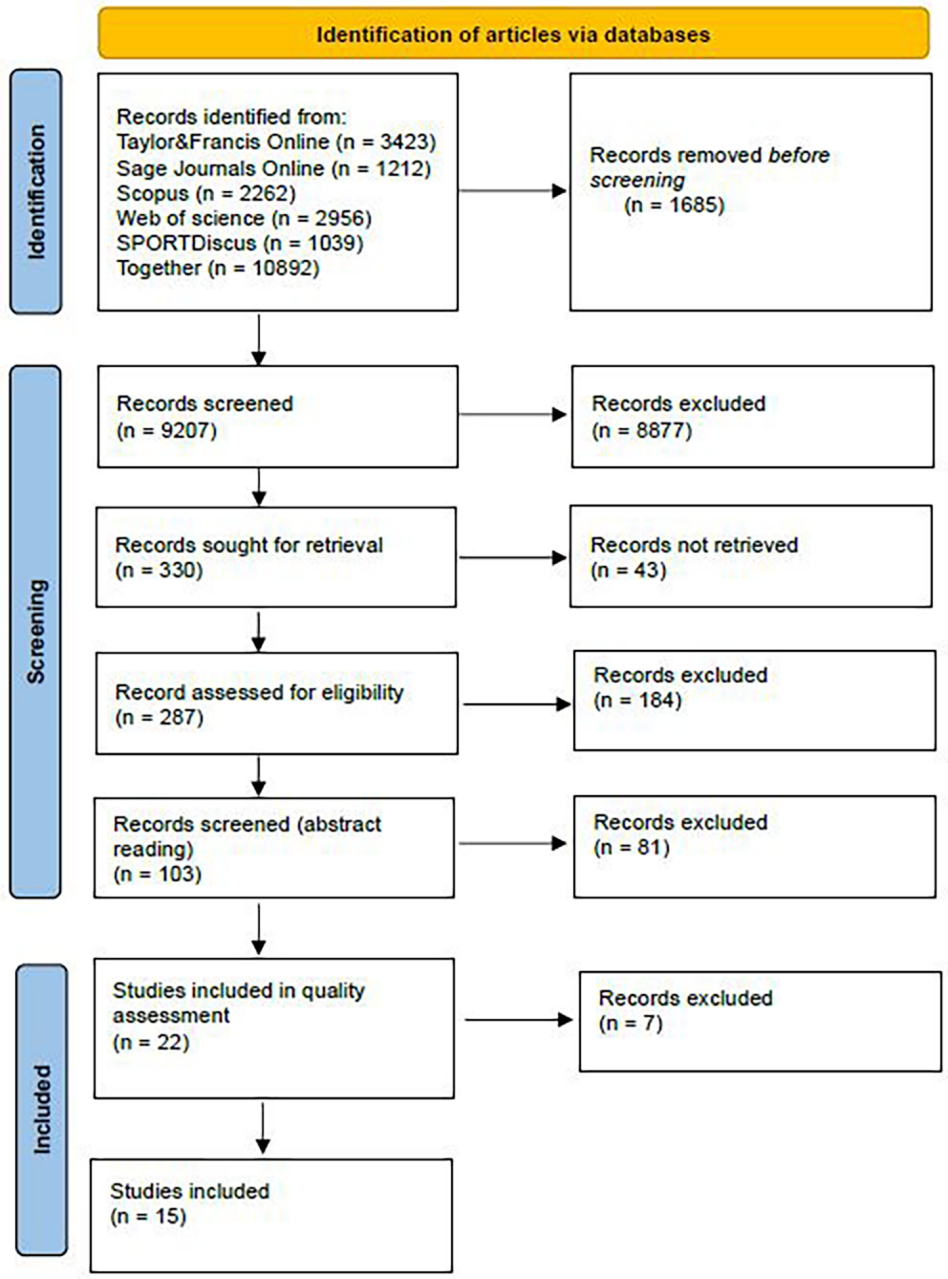
PRISMA flow diagram.

To search accurately and comprehensively for articles suitable for our objective, we followed the PRISMA flowchart (20), a diagram for systematic literature reviews, shown in Figure 1. Our main objective was to find articles addressing the impact of the coronavirus pandemic on different aspects of elite/professional athletes’ lives from micro-, meso- and macrosocial perspectives to derive recommendations for possible future encounters of the sport sector with potentially related situations.

### 2.1. Search strategy and identification of relevant papers

The search term we used to search the databases (1) Taylor&Francis Online, (2) Sage Journals Online, (3) Scopus, (4) Web of science and (5) SPORTDiscus for the period between (March) 2020 and (November) 2022 was as follows: (“athletes” OR “elite sport” OR “professional sport” OR “sportswomen” OR “sportsmen” OR “sport”) AND (“pandemic” OR “covid-19” OR “coronavirus” OR “SARS-CoV-2” OR “post-covid”). A total of 10,892 hits were identified in the databases selected. Due to the restriction of two databases that only allow screening of the first 2,000 hits, our selection was reduced to 9,207 hits. In conducting the screening, following our primary focus, we eliminated duplicates as well as articles mainly from the fields of psychology (e.g., research on mental health issues), physical education, physical activity and exercise in general, and sports medicine (e.g., injuries). In this way, we narrowed down our database of potential articles suitable for reading to 330 articles, 43 of which were not retrieved because the institutions’ catalogue of information resources did not allow authors to do so.

In a more detailed review of the remaining 287 articles, further exclusion criteria were established in mutual communication between all authors. Topics not included were the following: articles addressing the potential home field advantage without spectators; articles on fans during the pandemic; articles on virtual sports/esports; articles on the impact of the pandemic on the sports industry, business, sports facility maintenance and fan relations; articles on the impact of the pandemic on sports journalism and media; articles addressing the impact of the pandemic on individual brand management and team/league marketing; articles on the challenges for parents and youth and university sport; articles on the Tokyo Olympics; articles on health issues related to the pandemic (vaccination/masking/social distancing and debates) and on event virtualization and organizational issues during the pandemic; articles on the impact of the pandemic on grassroots and recreational sport; articles on philosophical issues (e.g., ethical and moral dilemmas); articles on the impact of the pandemic on referees and match officials; articles on the political use of sport and comparative studies. In this way, 103 articles were identified for which we performed additional screening by reading the abstracts.

### 2.2. Evaluation of the content and quality assessment of papers

Since we wanted to find and isolate articles dealing with the cross-section of the coronavirus pandemic and various (micro-, meso- and/or macrosocial) aspects of elite sport, as well as to formulate some recommendations for the sport sector on how to deal with similar circumstances in the future, the abstract reading focused primarily on identifying articles containing such recommendations. In this way, we eliminated a further 81 articles and arrived at a selection of 22 articles that were included in the quality assessment. To assess the quality of the 22 remaining records for final inclusion, analysis and synthesis, two authors independently conducted a quality assessment of the selected papers, following the assessment tools for qualitative studies as defined by Hawker et al. (21). Nine assessment questions (“abstract and title”, “introduction and objectives”, “method and data”, “sampling”, “data analysis”, “ethics and bias”, “results”, “transferability or generalizability” and “implications and usefulness”) were answered using four ratings (“good” if the information was complete and clear; “fair” if the information was not complete or clear; “poor” if there was minimal or unclear information; or “very poor” if there was a lack of relevant information). A comparison of the two authors’ ratings led to the agreement to additionally exclude 7 articles (either because they focused on psychological or medical aspects or because their conclusions cannot be generalized due to the specific context).

## 3. Results and discussion

The Covid-19 pandemic affected all sectors of society and various aspects of our lives, but its effects were not experienced or felt with the same intensity everywhere. As Rowe (5) wrote, professional sport felt the impact of the pandemic particularly intensely, not so much because of its global interconnectedness, which is not specific or unique to sport, but because of its reliance on commercial, media-funded live events that take place in front of a mass audience as a necessary aspect of the spectacle for another, much larger, distant audience.

The main findings of the review of the selected articles, which are also summarized in Table 1, can be roughly divided into three groups that also overlap: (a) Inequalities—articles that started from existing inequalities in sport (e.g., gender, ability, age) and addressed their exacerbation in the context of a pandemic; (b) Crisis management—articles that saw the pandemic as an opportunity to identify key factors and aspects that should be considered in moments of social disruption; and (c) Opportunities—articles that saw the pandemic as an opportunity for a new beginning, a potential transformation of sport into a more equal, socially responsible and environmentally friendly institution. The three groups were formed after the initial coding process and using an inductive process by which we attempted to capture the foci of the reviewed studies as effectively as possible from the bottom up, organizing the data into increasingly abstract units of information.

**Table 1:** A brief summary of the included studies.

Reference	Country	Aim of the study	Main findings/conclusions	Classification
Bowes et al. (8)	UK	To consider the perceptions of elite female athletes on the state of their sport prior to Covid-19 and the impact of the pandemic on their sport	Four main themes: questioning the ‘boom’ narrative of women’s sport (despite progress, women still feel undervalued in the sporting world, especially in terms of media coverage and funding), financial insecurity (inequalities in prize money, sponsorship, salaries, full-time professional status), media coverage issues (despite progress, off-peak coverage; reliance on social media), consideration of positives (potentially increased focus on other aspects of sport; for support of women's sport; notions of a fresh start)	Inequalities opportunities
Bowes et al. (7)	UK, Ireland, New Zealand, USA	To consider the impact of the Covid-19 pandemic on elite female athletes by examining the impact of the lockdown on training, welfare, finances and the immediate impact on women’s sport	The immediate impact of the lockdown on female athletes: financial (in)security (funding, salary, sponsorship were affected), training practices (amount of training reduced, type of training changed), engagement with coaches (support; importance of digital technology; some did not have enough mental support), immediate impact on training in women's sport (the experience was different for women—gender imbalance, return to play, access to resources as a secondary priority for women's sport); the virus did not cause the inequalities but exacerbated them	Inequalities crisis management
Clarkson et al. (9)	Australia, England, USA	To examine federations’ responses to Covid-19 through case studies of women’s football	Gender inequalities in return to sport; different responses to Covid due to different historical, political and social contexts—deep-rooted social structures as dominant institutional logic, constrained environments (influence of social and cultural values) and governance structures as explanatory variables for differences between NFA actions; importance of (clear crisis) communication for reputation management; importance of working with stakeholders to support gender equality	Inequalities crisis management
Pape and McLachlan (10)	Not specified	Using the pandemic as an opportunity to reflect on how the ideology of dependency hinders women’s place in sport and how the notion of interdependence might help reshape the unequal landscape of sport post-pandemic	Pandemic could disproportionately affect women's sport; pandemic exposes myth that men's leagues and clubs are independent; opportunity to reflect on sport: focus on interdependence exposed by pandemic; potential of turning pandemic into an equalizer (incorporating diverse women's voices)	Inequalities opportunities
Hu et al. (6)	USA	Examine the status of the sporting identity of Paralympic participants and how it has been affected by the Covid-19 pandemic	Paralympic athletes report strong athletic identity compared to non-disabled athletes; identity multiplicity is an important factor in avoiding AI difficulties during disruption (loss of identity correlates with depression and anxiety); complaints about inequalities in funding between Paralympic and Olympic athletes; impacts of pandemic on AI: challenged, unchanged, strengthened (using pandemic as an opportunity)	Inequalities crisis management
Tjønndal (11)	Norway	To investigate how the pandemic affected athletes’ training and competition activities (focus on the use of digital technologies)	Changed training habits (reduced training load; increase in online training, decrease in training in sports facilities); well-being (concern about performance, lower motivation; feelings of isolation); general agreement with restrictions; women and athletes from urban areas are more likely to participate in online training; athletes from individual sports are least likely to participate in digital training; male and older athletes are more likely to participate in online competitions;	Inequalities crisis management opportunities
Rowe (5)	Not specified	To reflect on sport during the pandemic with the help of a sociological approach	Pandemic has shown that sport is potentially vulnerable because of its global interconnectedness; as part of capitalism, sport has produced various inequalities (e.g. class, race, gender, sexuality, age, ability), so less lucrative and already more disadvantaged sports (e.g. women, disability, semi-professional sports) received less attention during the pandemic—the virus exacerbated existing inequalities created by the commercialization and hierarchization of sport; the concept of cultural citizenship could be mobilized to strengthen more egalitarian and inclusive practices in sport	Inequalities opportunities
Grønkjær and Frøyen (12)	Norway	To understand how the leaders of the national women’s handball team perceived and exercised their leadership role to sustain performance during the Covid-19 pandemic	Regular communication is crucial in a crisis; to describe the team's leadership and management processes during the pandemic: digital communication (increase in digital meetings, higher level of collaboration, time efficiency), flexible management (ability to expand players’ technical and physical training and game tactics, individual plans for players; psychologists available for each player; need to think of limitations and potential risks in the future) and collective leadership (players, leadership and management; relationships contribute to trust and confidence that decisions are safe and in the ‘ best interest of the players)	Crisis Management
Costa et al. (15)	Italy	To explore the experiences of coaches’ and athletes’ in adapting goal setting during the first pandemic lockdown	Three main themes on goal setting (reviewing goals and moving towards new ones, letting go of goals, trying to hold on); changes in goal setting affect emotional, cognitive and performance consequences; ability to change goals correlates with ability to overcome failures, follow-up and goal evaluation should be done throughout the season; from the perspective of care ethics, it is important to prioritize differently on occasions like a pandemic	Crisis management
Washif et al. (14)	Malaysia	To investigate the perceptions of elite athletes’ on quarantine training camps	The quarantine training camp can reverse some harmful effects of situations like the Covid-19 pandemic—training routines, performance support and perceived stress improved during the training camp, nutritional choices, mental well-being, emotional well-being and training motivation improved	Crisis management
Whales et al. (13)	Australia	Researching the Covid-19 pandemic from the perspective of professional athletes	After the closure of the netball league, the unprecedented situation led to more meetings (collective leadership and decision-making was valued by players—inclusion fosters trust); access to technology facilitated their training commitments and enabled continuous communication (more necessary in times of uncertainty)	Crisis management
Glebova et al. (16)	Not specified	To identify, describe and visualize the transformation process in sport during the pandemic Covid-19	In addition to challenges (e.g. planning training), there were also opportunities (e.g. improving environmental impact or improving and developing means of communication); the transformation of sport took place on several levels (main dimensions: psychological, social, modifications (time changes, policy changes), changes in demand (technology-based products and services));	Crisis management Opportunities
Thorpe et al. (19)	New Zealand	To examine how women (athletes, coaches, fitness instructors, studio owners) redefined their well-being during the pandemic	Wellbeing became known as a relational and non-linear state of becoming through connections with bodies, objects and the environment (closeness, technology, relationships of care); re-imaginings of life beyond narratives of health and wellbeing, towards becoming with the world; athletes worrying about their athletic futures; orientations of ‘letting go’ and ‘holding on'	Opportunities
Leng and Phua (18)	Not specified	To consider the role of athletes as role models in a crisis like the Covid-19	In various social disruptions, athletes—like all other public figures—could set a good example for their fans and encourage appropriate behavior, their behavior on and off the field can influence others	Opportunities
Wynes (17)	USA, Canada	To analyse emissions from team air travel in 2018 and 2020 to demonstrate the extent to which measures taken during the Covid-19 pandemic can be maintained to permanently reduce emissions from air travel in the sector	pandemic led to a number of procedural and schedule changes—action by the four major leagues would reduce emissions from air travel, additional action could extend the reduction even further; value of the sports field as a climate messenger, benefits to player health and performance	Opportunities

### 3.1. Inequalities—the deepening of already existing inequalities in sport

Sport is part of the capitalist way of organizing social life. As a product of capitalist logic (22), it has itself produced various inequalities during its development (class, gender, ability, sexuality, age, race, etc.), which have deepened and become potentiated in the context of the pandemic. Not everyone was in the same boat—even though the virus could potentially infect anyone, regardless of their personal circumstances, that does not mean it was common as a problem or that we all faced it in the same way. Less lucrative and already more disadvantaged sports (e.g., women, disability, semi-professional sports) received less attention during the pandemic [(7); see also (9)]. Taking the experiences of female athletes as an example, despite perceived progress in the treatment of women’s sports, female athletes reported that inequities still existed [e.g., in media coverage of women’s sports compared to men’s sports or at the level of financial (in)security] (7, 8). The pandemic also created a situation in which women’s and men’s sports were treated differently in terms of access to various resources and efforts to restart the sport—women’s sports were not a priority (7, 9, 10). In addition to the differences between women’s and men’s sports, it seems useful to point out the differences between sports as well as within a given sport. For example, analysis of pandemic responses in the context of women’s football has shown that there are differences due to historical, political, and social factors (9). In a study that examined athletes’ participation in online training and competition, there was not only a difference in coping strategies by gender, but also by age and place of residence (11).

### 3.2. Crisis management—success factors in times of social disruption

The Covid-19 pandemic also sharpened and brought to the forefront the factors that were important in managing this situation and that are important in managing situations related to such social disruptions. At the crisis communication level, the importance of stakeholder collaboration (9) and collective leadership (12) emerged. Specifically, athletes reported valuing approaches that include them in meetings (13), listen to them, and communicate and collaborate with them regularly (12). Communicating with athletes builds trust, and that is key to believing that actions are taken in the best interest of the players. Flexibility at the training level also proved important for coping—athletes’ training habits changed during the pandemic (11), in addition to individual training adaptations (12), so-called quarantine camps (14) improved training routines, performance support, dietary choices, training motivation, and emotional well-being. Also positively impacting coping with lockdown, various interruptions, and limitations, is a multiple identity challenge rather than a fixation on a purely athletic identity (6) and flexibility at the level of goal setting. The latter pointed to the need for follow-up and goal evaluation throughout the season (15). None of this, e.g., successful crisis communication and adaptation at the level of training and goals, would be possible if we did not have the appropriate technology. During the Covid-19 pandemic, digital technologies were key to successful adaptation of training, training commitments, communication between players, coaches, and management structures (7, 11–13, 16).

### 3.3. Opportunities—the pandemic as an opportunity to transform sport

Several articles highlight not only some of the limitations and negative effects of the pandemic on sport, but also the attitude that saw the pandemic as an opportunity for a new beginning and various changes in the sport sector. On a broader societal and institutional level, the pandemic demonstrated that sport is globally interconnected (5), debunked the myth of independence of men’s leagues and clubs (10), and thus could become a catalyst for addressing various inequalities in sport, such as gender inequality (8). In this context, Rowe (5) highlighted the concept of cultural citizenship as a possible way to initiate and strengthen egalitarian and inclusive practices in sport. Various authors also highlighted the possibility of developing communication tools (16) or, for example, using the pandemic as an opportunity to transform sport into a more environmentally friendly activity (17). If, on the one hand, sport could become a “role model” at the level of respect for the environment and striving for sustainable development, athletes could be role models in times of social disruptions at the level of their behavior and adherence to social rules (11, 18). On a more individual level, the pandemic was also highlighted as a situation in which some athletes became aware of their well-being as relational and nonlinear, a state of becoming through relationships with bodies, objects, and the environment (19). From the perspective of the ethics of care, the situation enabled the reordering of individual or institutional priorities (15).

## 4. Draft guidelines

If we try to translate the lessons and findings of the authors of the selected research articles into the three groups highlighted above, or derive from them guidelines for the behaviour of athletes in potentially similar social situations in the future, we can first say the following: the guidelines we present in this paper are rather common sense due to the desire to generalize them as much as possible, although we do not want to diminish their value. Indeed, they are the result of research and research approaches that provide us with supporting empirical evidence and have not been produced on the basis of (more or less probable) speculation about how the pandemic (and possibly similar situations in the future) has affected and impacted the lives of elite athletes. In this respect, they provide more tangible and informed starting points and advice for action in the context of similar societal upheavals.

In the broadest sense, i.e., at the structural and macro-social level, it seems worthwhile to re-emphasize the inequalities that the virus made visible and reinforced but did not cause. Sport is not immune to various inequalities that also exist at the level of broader social life and are reflected therein through gender, race, ability, class, sexual orientation and the like. In this respect, probably the most far-reaching and radical lesson from the pandemic is the need to change sport, sport institutions and sport structures in ways that lead to greater equality, equity and inclusion. Equitable sport structures are not only important from the point of view of increasing the participation of different social groups in sport, but they also need to be considered at the level of the relationship between the world of sport and the environmental dimensions. These are efforts that affect the entire sport complex and all its actors, although the most tangible and effective achievements in this context will come from those actors and stakeholders who can work effectively at the level of potential change at the fundamental institutional level and at the level of the key structures of sport. Sport as a social and global institution has been exposed as vulnerable by the pandemic, but not in the sense that everyone is equally vulnerable. Efforts to strengthen its resilience must therefore first and foremost keep in mind the values of equality, mutual respect and care for fellow human beings as well as for the planet, which, if we continue with “business as usual”, i.e., if we do not take seriously the predictions of scientists and aim for sustainable development and sustainable social subsystems (including sport) together with a human-friendly environment, we will also wipe out the possibilities of everyday life as we know it. As Rowe [(7), p. 710] notes, “Critical sport sociology has constantly questioned the skewing of social structures, values and practices by ’sportainment’s’ addiction to economic capital growth at all costs.” It is time for more radical and responsible responses that have a transnational and intergenerational sensibility.

So the most comprehensive guideline or starting point for the future is:

• Awareness and appeal to those responsible for the necessary and radical changes in the existing organization of the sport sector and the existing sport structures, which should be based on equality and sustainable development and responsibility towards the environment.

The meso-social level is the level of individual sports organizations, in the context of which the selected studies highlight the importance of quality and effective crisis communication. Sport organizations confronted with a new situation survived the period of uncertainty more easily if they were able to

• Work with stakeholders and adopt management principles based on collective leadership, i.e., a form of leadership based on pronounced democracy and mutual cooperation.

In addition to the forms of cooperation between the fundamental actors of sport and their leadership, the relationship between sport organizations and individual athletes has proven to be particularly important:

• Regular communication with and information for athletes, whereby it is necessary to involve athletes in mutual dialogue, to give them a voice and listen to their needs, and in this way to build mutual trust and respect. In this sense, the pandemic has reinforced the need to integrate athletes’ values and the athlete-centered coaching approach that is already an established practice in the coaching context (23).

The organizational meso aspect is often inextricably interwoven with the micro-social aspect, i.e., an aspect of the athletes themselves, their behaviour in the context of the pandemic, their more individual adaptations on the one hand and adaptations requiring relationships with others on the other. The athletes pointed out that when their previously accustomed daily lives were interrupted, other sensitivities and states of awareness sometimes came to the fore, which could serve as legitimization for other priorities during this time. In the case of a more serious and unavoidable interruption of the training process, the individual athlete benefits from:

• Multiple identities, which provide the individual with a protective umbrella and support in other identities when the sporting identity is interrupted (for example, dual-career athletes can focus on more intensive pursuit and progress at the level of the school/professional identity during the interruption of training and other commitments related to the sporting identity).

Athletes who can pursue their sporting interests even in a new social situation will find it easier to pursue their goals if their emotional well-being is taken care of and they receive general performance support, nutritional choices and motivation from coaches, and if, in addition:

• Training is made less rigid and more flexible and adaptable (including through the innovative use of technology and other technological tools), both at an individual level, i.e., at the level of the individual athlete, and at a more group and team level (e.g., with greater distance from the rest of social life and the introduction of special training camps)
• Athletes are also allowed and even encouraged to be more creative and flexible at the level of expected results and goals (here a review of results and goals is expected during the season).

## 5. Summary

Top athletes can be understood as a specific social group that had to face and adapt to the newly created situation in a special way to overcome the obstacles that appeared during the pandemic. Moreover, sport is such a broad field with different practices and discourses that it is almost impossible to speak in general terms about the impact of the pandemic on athletes and how to adapt to it. For example, the runner, who—depending on the time and place—was able to continue her outdoor training relatively unscathed during the pandemic, on the one hand, and the swimmer, who required a swimming pool for her training, on the other, were certainly not in the same situation. Nor was the affluent male NBA athlete who was able to continue his training in his home gym in the same situation as his female counterpart, who was generally much less likely to find such supportive conditions. More similar comparisons could be made, highlighting both the more striking differences and the usually less exposed but more important intersections, but we have focused on more general and common uncertainties and changes in this paper.

At the broadest, i.e., macro-social level, the multiple inequalities that have been included in the organization of modern sport since its inception are highlighted as key, and its fundamental structure should be thoroughly and all the more reconsidered and changed, especially in contemporary times -not only because it is very conservative and exclusionary in some of its structures, but also because we are living in a time of great ecological change, to which the existing structures and forms of social organization in elite sport are currently unreceptive. Equality and sustainable development are key at this broadest level.

The meso-social level as well as the level of individual sport organisations gave us an insight into the need for effective crisis and quality communication involving different stakeholders in sport (including regular communication with individual athletes) and based on collective leadership. Finally, at the micro-social level, we highlighted the athletes themselves and, on the one hand, the importance of having multiple identities grounded in multiple roles and sources of support, caring for their emotional well-being and other forms of support (motivation, nutrition) and, on the other hand, the need for flexibility and adaptability in both their training and expected outcomes and goals.

## 6. Implications for the future

While it may seem that the pandemic of the early 2020s took us by surprise and unexpectedly, it is probably more accurate to understand it as a process of discovery and adaptation to what was hiding behind the facade of “normalcy” that had been taken for granted for decades: The coronavirus was “a ‘revelation’ in the original sense of the word ‘Apocalypse’. A sort of an apocalyptic x-ray that has unveiled not only what the scientists were persistently warning of for decades /…/ It also unmasked a global system based on vicious circle of extraction, exploitation and expansion” [(4), p. 8–9). If we do not question this level of “normality”, then the Covid-19 pandemic is not so much something we may have already left behind, but rather something we can see as a prediction of future pandemics that will primarily affect those who are already worse off in the existing order, which is characterised by various inequalities.

The Covid-19 pandemic has highlighted existing social inequalities, gender, racial, class and other divisions that have been inscribed in the prevailing social order and, by extension, in its subsystems. In this paper, we have looked at one aspect of the social subsystems, namely elite sport, which was affected by the pandemic.^1^ In the context of sport, at the intersection with the pandemic, many aspects worth exploring have emerged—from the more direct impact of the pandemic on the organisation of competitions and sporting events, the financial impact of Covid-19 on the functioning of sports organisations and clubs, the media and communication practises related to sport, the establishment and management of new forms of exercise and physical activity, the consumption of sport when live consumption of competitions has been banned, the symbolic questioning of the role of sport in society, etc. Since we did not focus on these issues in this article, but were primarily interested in the impact of the pandemic on elite athletes and how they deal with it, these could be possible starting points for further research. Indeed, all of these are important aspects of modern (elite) sport that—in the face of an increasingly uncertain future—deserve all the more to be addressed in depth or through similar reviews.

Social situations, such as the one triggered by the Covid-19 pandemic, are situations that, at least superficially, disrupt our taken for granted and familiar routines. The guidelines we have derived from the research findings during the Covid-19 pandemic are neither a magic solution nor definitive answers to the question of how to survive similar situations relatively unscathed. Finally, each new crisis has its own peculiarities and therefore requires specific adjustments. Nevertheless, we hope that the general guidelines outlined here can defuse the anxieties that arise in the face of unprecedented challenges. Ultimately, however, it is the structural aspects that need to be addressed most thoroughly—after all, these can minimize the possibility of new major disasters.

## Data Availability

The original contributions presented in the study are included in the article/Supplementary Material, further inquiries can be directed to the corresponding author.

## Footnotes

^1^Our decision to focus on elite sport was, in part, influenced by the personal and commercial stakes associated with it. However, there is also a need to synthesize research on recreational and youth sports. They are organized and integrated into social relationships and personal lives in ways that differ from elite sports. For this reason, the impact of the pandemic was experienced differently in sociological terms.
